# Modified teaching approach for an enhanced medical physics graduate education experience

**DOI:** 10.2349/biij.7.4.e28

**Published:** 2011-10-01

**Authors:** IB Rutel

**Affiliations:** Department of Radiological Sciences, College of Medicine, University of Oklahoma Health Sciences Center, Oklahoma City, United States of America

**Keywords:** Medical physics education, guided discussion, problem based learning

## Abstract

Lecture-based teaching promotes a passive interaction with students. Opportunities to modify this format are available to enhance the overall learning experience for both students and instructors. The description for a discussion-based learning format is presented as it applies to a graduate curriculum with technical (formal mathematical derivation) topics. The presented hybrid method involves several techniques, including problem-based learning, modeling, and online lectures, eliminating didactic lectures. The results from an end-of-course evaluation show that the students appear to prefer the modified format over the more traditional methodology of “lecture only” contact time. These results are motivation for further refinement and continued implementation of the described methodology in the current course and potentially other courses within the department graduate curriculum.

## INTRODUCTION

Teaching methods to increase student understanding and enhance the learning experience have evolved throughout history. The methods span a spectrum of student involvement, from passive spectator to active educator, and have been given names like “Socratic Method”, “Modeling”, and “Lecture”. One might postulate that the underlying question always remains the same: “What is the most effective way to facilitate the increase of a student’s knowledge?” Ideally, and with an infinite amount of time, the instruction would best be handled through a one-on-one basis with understanding of prerequisite topics being assimilated prior to continuing the instruction. However, the question posed above must be modified to include the realities of limited resources and might now read: “What is the most efficient method to promote effective facilitation of class learning?” Arguably, this now includes the challenge of increasing the specific (subject material-based) knowledge of a class (multiple students) over the given time allotted by the programme or time permitted for the instructor. This maxim is stated as the motivation for the introduction of a modified instruction method, as an instructor grapples with the challenges of teaching a small- to medium-sized class about a technical topic (or series of topics) within the period of one semester.

Recent history identifies the predominant use of lecture-based teaching formats, where the instructor stands at the podium, computer or dais, and imparts the subject material over the course of an hour or more, with the intent that an interpretive elucidation of a text will clarify concepts and enhance the retention of the material. This produces the most passive form of student involvement, namely, as the spectator. This method may prove to be efficient but may not be as effective as an instructor might desire [1]. Reasonable retention may be achieved by the students, but the content is limited to the instructor’s choice of topics. Furthermore, the “conversation” will only deviate from the lecture plan by small amounts to accommodate questions from the audience, as there is a tendency to keep deviations to a minimum in order to complete coverage of the required material. If the lecturer is good both as an instructor and an orator, this method should provide adequate coverage of the subject matter, allowing the students to be exposed to the appropriate material and providing a modicum of enjoyment to help enhance the knowledge uptake. While many in the education profession would like to believe that they are excellent instructors, it would be remiss to think all instructors are excellent orators, leading to moderate or poor (and perhaps frustrating) experiences for both the lecturer and the students, alike.

The following case study stems from such a frustration, in an attempt to provide adequate coverage of a technical (mathematically rigorous) course, while enhancing the overall experience for both the instructor and students. The method and course were implemented at the University of Oklahoma Health Science Center (OUHSC) during the Spring semester of 2010. The graduate course covers the theory, implementation, and practical applications of radiation measurement and detection. The course is technical in both the concepts and descriptions, including differential equations and solutions, statistics, electronics theory, detector design and theory and signal analysis. The specific challenges were to increase the student participation, maintain course material coverage, provide a stimulating environment for discussion and ensure that knowledge of the course material was assimilated.

The methods presented in this paper focuses on the implementation in a medical physics graduate course setting. It should be emphasised that the format of the course can be ported to the instruction of residents; this is also being implemented for radiology residents in our department, but will not be the focus of this discussion. The context of the course has been specifically presented to indicate the level at which the students are expected to perform, but this admission should not detract from the understanding that the methodology may work equally as well in other technically (or mathematically) rigorous courses.

## METHODS

The choice of instruction method was carefully considered, but the desire to remove the primary lecture during class contact hours was a key point of modification. Various methods were investigated, including “Modeling” [2–4] and “Socratic Method” [5–7]. Modeling is a method being implemented at both the high school and undergraduate levels for physics, where a physical concept is investigated through demonstration, laboratory investigation, simulation, and finally theoretical derivation. Although this technique makes sense for courses that introduce several specific concepts with reasonably easy demonstrations, it was determined that it is difficult (and overly time-consuming) to have the students explore each of the detector designs and their responses to various radiation sources, and finally derive the models for signal generation. As interactive and exciting as this method is, it did not seem feasible in the graduate course setting due to the depth of understanding and difficulty of derivations required, which shortens the amount of time available to cover the breadth of material that needs to be presented during the semester. Although this method works well for the small class sizes anticipated for the course (7–9 students), it was not chosen due to the time concerns listed above.

The “Socratic Method” is a commonly implemented teaching method in graduate school, but for topics and studies which are generally concept-based, and in large classes, i.e. law [8, 9] and medicine [10–12]. In large classes, it is generally easier to find students with the courage to speak up and answer the questions initiated by the instructor. This in turn can lead to further class participation through continued interest and expansion of topic discussion promoted by students’ answers or further clarifications, probing and follow-up questions by the instructor. This method is promising, although there are challenges in covering the technical equations and derivations without resorting to slides or blackboard lecture derivations. The anticipated class size is also worrisome, since smaller classes are less “anonymous” and tend to deter the active involvement of students and continued discussion. A further comparison of the Socratic method, lecture, and a personal instruction method can be found in [13].

Introducing the topics of medical physics education at an undergraduate level or high school (secondary) level has also been investigated with the production of computer-based modules [14]. The tools presented in the literature have been used to provide a heuristic overview of medical physics as a profession and used medical-based examples to illustrate physical concepts. Pre-recorded lecture experiences (modules and recorded didactic education) for students are a new method; however, the use of such aids is generally reserved for ancillary or extra topics and not as the sole source for didactic lectures, as presented here.

A hybrid method was selected to accommodate a modified version of the “Socratic Method” learning style (referred to here as guided discussion), the concept-investigational attributes of “Modeling” in undergraduate physics through the use of one of its tools called “white boarding”, and the introduction of online lectures to ensure course material coverage. The intention for this implementation is to provide a positive and cooperative environment for student participation; an adherence to the more classical argumentative style of the Socratic Method was determined to be too confrontational for the smaller class sizes.

To ensure participation, a substantial portion of the student’s evaluation was based on the subjective evaluation of their participation (30% of their entire grade). The interesting part about the participation grade in this model is that there is also a component of evaluation during discussions, since the questions asked and answered by the students allows for a unique opportunity to determine their depth of knowledge through conversation (if the instructor wishes to add this to the grade determination). So the inclusion of a participation grade need be neither obligatory nor perfunctory and can truly be a measure of the students’ performance in this proposed method.

The online lecture preparation began with the original slides used in previous iterations of the course, coupled with a recorded instructor voice overlay. Although this portion of the method is not required (and may be time-consuming to finish), it allows for a “safety net” for the instructor, ensuring that an instructor interpretation of the required material is available to the student (whether they choose to watch the online lectures or not). In this implementation, the online material was produced using Adobe Captivate, a Shure Microphone (model SM58) and a Tascam USB 2.0 MIDI interface (preamp/computer interface) model US-144. Focus was given to choosing recording equipment that produces good audio quality recordings and professional-sounding voice-overs. The Captivate software has some unique features which allow pausing of the recording, viewer interaction and choice of instruction speed, and an interactive experience on images/displays where further details may be provided by employing “mouse-overs” (pop-up descriptions when the cursor is placed over a specific area). A full description of the Captivate software can be found at (http://www.adobe.com/products/captivate). The final interactive product is output in a Flash format; unfortunately, this may be problematic for those using hardware which does not allow Flash playback. The finished lectures were uploaded to a learning management software package (Blackboard), and could be accessed only by participants in the course. It should be noted that the use and/or posting may require copyright approval from the publisher of the text if utilising images captured from the text itself.

## EXPERIENCE

Preparation is required for both student and instructor utilising this method. The student should be prepared for a detailed discussion about the specific subject material to be covered during the specific contact time. To this end, the student may have prepared by watching the online lectures, reading the textbook, using outside resources to answer their own questions, etc. This preparation is highly dependant on the motivation of the student to prepare for a subject before the discussion period.

The instructor prepares by finishing the online recording (hopefully well in advance of the discussion session, allowing ample time for students to view), reviewing the text, and preparing a list of discussion topics or questions to facilitate the expected discussion, identified as a “road-map”. It was found that questions were a better choice for the road-map, since the preparation of the questions could be revised to probe a specific aspect of the material, or facilitate the next general topic to be discussed in a more fluid conversational weave. The less thought required in formulating the next non-sequitur question allows for a better focus by the instructor on the student responses, leading to more tailored (student- and topic-specific) questioning about the current discussion topic.

Prior to the class, both instructor and students should have completed the preparatory homework to ensure reasonable familiarity with the topics to be discussed. The class then gathers in a geometry suited for a group discussion (a quasi-circular configuration was employed). The time allotted for this class was 1.5 hours twice a week, which allowed for slightly less pressure in fitting the entire discussion into a customary one-hour period (the original lectures were presented in hour increments).

The contact time for discussion consists of four components, announcements, initial questions, topical discussion and exercises. Leading into the discussion with announcements about administration (homework assignment, test scheduling, and general class issues) helped to focus the class on the course and prepare the students’ attitudes for contribution. Keeping announcements reasonably short and maintaining a positive manner helped to set an appropriate tone to encourage participation.

The second portion is a general question-and-answer session. It was determined that some time at the beginning of the discussion should be allocated to permit the students to bring up questions they have generated from the reading. It helps on several levels to employ such an activity; the students have ample opportunity to ask their specific questions, the instructor can utilise the questions as a gauge of what may require more attention during the focused discussion time and there is a perceived enhancement to the sessions, where the students are given the feeling that they have control and dedicated time to inquire about topics at whatever level they choose, without having to attend office hours. The last aspect is also an efficient use of time for the instructor since one student’s question may spark another question from other students, or answer a question several students have about concepts or clarifications in the text. The time for this activity ranged from 0 to 30 minutes depending on the topics and the student’s preparation and/or understanding.

The next portion is the focused discussion period. During this time the instructor asks the questions from the road-map and leads a conversation with all the students. Having students answer questions during a lecture format has one major impediment: the lecture is the dominant form of communication, and the students are set into an abnormal state by having to answer questions. This will tend to decrease the students’ participation and limit responses to cursory answers. In the modified format, the new “normal” is to answer questions, so the students tend to have less of an impediment in answering. There are still pauses (sometimes long pauses) in response waiting times, but these cases were generally due to poorly-worded questions by the instructor, or lack of preparation by the students. Either of these causes can then be learning experiences for either the instructor or the student: rewording of the question, or asking a slightly different question would generally spark recognition of concepts in the class and the discussion would continue. It is not claimed that this technique provides a flawless method for fluid (non-stilted/unforced) conversations, but the new established normal for the students in class was participatory response, not silence.

Addressing the illumination of a complex derivation/equation moves us to the introduction of the whiteboarding exercise. Calculation exercises were compiled during the instructor’s preparation and introduced at appropriate (material-pertinent) times during the discussion. For example, a calculation for some property of scintillation detector response may depend on the materials used in the detector itself, so the class is broken into several teams and asked to calculate the response based on several detector material properties. The class groups are then given time (5–20 minutes) to work with the equation and determine the difference in the response. Groups can all be working on the same problem, or the problem can be split into multiple parts and each group can be given a portion of the problem to solve. A spokesperson from the group is then asked to present the group’s findings to the rest of the class. In general, the spokesperson should change with each exercise, to give each member of the group a chance to participate in the explanation. These exercises force the students to work with some of the equations in the text which may (or may not) be queried in the problems section of the text. If the instructor selects useful relations, then illustration of extreme conditions or varied inputs will add to the students’ understanding of the limits of the mathematical relation. The exercises have many advantages including instructor-guided problem-solving time, peer (group) problem-solving experience and opportunities to explore the complex mathematical descriptions of the course topics without slides or instructor-based presentation derivations. Presenting actual derivations will still be handled by the instructor, using blackboard presentation, unless the instructor has confidence that a group exercise will present the correct work within a reasonable amount of time.

The time required for each of these instruction sections during contact hours will depend on the subject matter and the students’ preparations. There was no need to set specific time periods, since there was no rush to finish the road-map questions. This removal of pressure is directly attributed to the accessibility of online lectures, and pressure to include topic coverage may again be present if alternate forms of the lectures are not available. This issue is greatly dependent on the instructor’s confidence in the students, the text and the ability of the students to determine the important points from the text, assimilating the pertinent knowledge.

## RESULTS

Assessment of the experience was performed with an end of semester survey. The questionnaire included questions on the usefulness of the online lectures and guided discussion, comparison to the equivalent “lecture” course (as evidenced by information provided in the online lectures alone), and ability of each portion to be sufficient in conveying the course material alone. The survey was based on a 0–4 scale with 0 = “not applicable (unable to judge)”, 1 = “not at all (rare/never, strongly disagree)”, 2 = “minimally (occasional, disagree)”, 3 = “generally (usually, agree)”, 4 = “consistently (often/always, strongly agree)”. The questions are listed in Table 1.

**Table 1:** Student evaluation questionnaire.

**1**	Socratic/online format provides acceptable environment for learning course material.
**2**	Socratic/online format conveys increased breadth and depth of course content over traditional lecture structure
**3**	Socratic/online format takes time from instruction better spent elsewhere (Please comment on where time should be spent)
**4**	Socratic/online format provides learning activities appropriate to course content
**5**	Socratic/online format enhances retention of course content over traditional lecture format
**6**	Socratic lecture uses an interactive style of teaching
**7**	Socratic lecture is better overall in comparison to the traditional lecture structure
**8**	Socratic lecture format stimulates enthusiasm and high standards for learning the course material
**9**	Socratic method facilitates independent thinking/problem-solving of course material
**10**	Socratic lecture is sufficient for learning concepts without online content
**11**	Socratic lectures respect residents/participants as individuals
**12**	Online content is presented in an organised and clear manner
**13**	Online content is presented at a level similar to or greater than the traditional lecture format
**14**	Online content is useful as a learning aid
**15**	Online content requires too much time to view (Please comment)
**16**	Online content is sufficient for learning concepts without lectures

**Figure 1:**
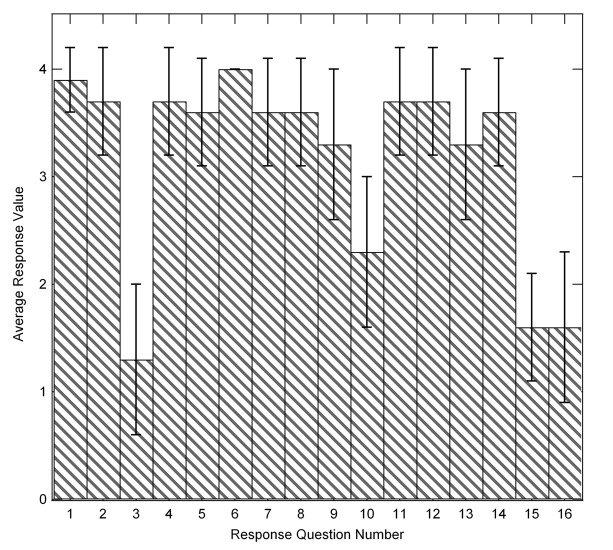
Average response values from student survey (n=7, student responses). Numbers indicate the survey question response being averaged. Error bars show the calculated value of one standard deviation for each response.

The sampling size includes seven (7) responses (the class size) and is summarised in Figure 1. The limited data show on average positive (high number) responses for most of the questions. Three questions averaged below a value of 2. Questions 3 and 15 are purposefully worded to give a low value for positive response, and question 16 (and to a lesser extent question 10) indicate the response to student perception: that neither the online content nor the guided discussions were sufficient alone to provide adequate information.

Problems with this survey include the lack of questions about the textbook and its role in preparing the student for guided discussions or overall assimilation of course content information. Potential questions about this resource would include the ability of the textbook alone, and in tandem with the rest of the provided content, to sufficiently convey the concepts and its ability to provide adequate information for the students. These questions were not included since the questionnaire was focused on changes in the format and use of a text is assumed to be standard in any graduate course. This oversight will be remedied in future surveys to establish a better understanding of how the text contributes to the overall information assimilation in conjunction with, and in lieu of, the instructor-prepared materials.

## CONCLUDING REMARKS

It is clear from the survey that the students were in favour of the modified format. Free form responses from the survey also provided support for the modified format, as well as the inclusion of more evaluations (like quizzes) to help the student find important concepts on which to focus (for which they may not have attributed enough time in their preparatory studies). Comments also referenced the increased amount of work required for this format but noted the resulting (perceived) increase in information acquisition. Out-of-class preparation was mentioned as a key component to feeling well-prepared for the discussions, and the discussions themselves were useful in elucidating concepts taught in the text. Finally, mention of the enhanced learning from other students’ perspective is also relayed, where the added value from a student-contributed viewpoint was a benefit and at times a better description than the instructor’s response.

From an instructor’s perspective, the preparation in using this format for the initial offering is time-consuming. The preparation of online lectures average 3–4 hours per lecture (27 lectures in all). The added preparation of the road-map and meaningful exercises could also take 1–2 hours, adding to an even larger time commitment. However, in future course offerings these times will be mitigated to correcting slides, modifying voice-overs, editing of poor exercises and focused discussion sections; reducing the amount of required preparation time for review of the text and the road-maps. This format requires large time commitments upfront with the benefit of reduced preparation times for future offerings.

The student access and discourse during the semester allowed evaluation of the student’s knowledge and understanding at a level not possible in previous years, where the only student evaluations were from tests, labs, and homework. The course evaluation for this semester included numerical scoring of tests, labs and homework with the added subjective scoring for course participation and a presentation. The presentation was an added chance for the students to use the synthesised information acquired during the semester and apply it to a novel detector system. The presentations were thoughtful and the descriptions incorporated the concepts learned throughout the course, forming a final evaluation to determine if the students could use what they learned and apply it to an unknown system; essentially, the presentation of acquired knowledge used in a novel way.

The introduction of this modified teaching method has provided a model for incorporating alternative (non-lecture based) teaching methodologies into a technical upper-level undergraduate, fundamental graduate level or physics-based technical radiology resident course. The students found the experience to be useful and generally positive, leading to a conclusion that this method should be employed in the future for the current course, and possibly expanded to other courses in the programme.

Although this paper is certainly only a preliminary study on the merits of this methodology, further research should and will be continued. The major message from this paper is that the lecture-based format may not be the most efficient way to teach upper-level students in technically rigorous courses. Exploration of new techniques and combination of techniques should be encouraged for all educators in similar fields and varying educational levels to provide a better service and experience for both the students and themselves. The methodology and experience provided here also proves that, in at least one implementation, a mathematically rigorous course (with reasonable depth and large breadth of technical information) can be performed without having to spend the majority of contact time orating a didactic lecture, when the time could be better used to facilitate the learning process more directly.
